# Prognostic Significance of Left Ventricular Noncompaction: Systematic Review and Meta-Analysis of Observational Studies

**DOI:** 10.1161/CIRCIMAGING.119.009712

**Published:** 2020-01-21

**Authors:** Nay Aung, Sara Doimo, Fabrizio Ricci, Mihir M. Sanghvi, Cesar Pedrosa, Simon P. Woodbridge, Amer Al-Balah, Filip Zemrak, Mohammed Y. Khanji, Patricia B. Munroe, Huseyin Naci, Steffen E. Petersen

**Affiliations:** 1 William Harvey Research Institute, NIHR Cardiovascular Biomedical Research Centre at Barts, Queen Mary University of London, Charterhouse Square, United Kingdom (N.A., M.M.S., C.P., S.P.W., F.Z., M.Y.K., P.B.M., S.E.P.).; 2 Barts Heart Centre, St Bartholomew’s Hospital, Barts Health NHS Trust, West Smithfield, London, United Kingdom (N.A., M.M.S., F.Z., M.Y.K., P.B.M., S.E.P.).; 3 Cardiovascular Department, Azienda Sanitaria Universitaria Integrata, University of Trieste, Italy (S.D.).; 4 Department of Neuroscience, Imaging and Clinical Sciences, Institute of Advanced Biomedical Technologies, “G. d’Annunzio” University, Chieti, Italy (F.R.).; 5 Imperial College London, Kensington, United Kingdom (A.A.-B.).; 6 Clinical Pharmacology, William Harvey Research Institute, Barts and The London School of Medicine and Dentistry, Queen Mary University of London, United Kingdom (P.B.M.).; 7 Department of Health Policy, London School of Economics and Political Science, United Kingdom (H.N.).

**Keywords:** cardiac imaging techniques, cardiomyopathies, meta-analysis, prognosis

## Abstract

**Background:**

Although left ventricular noncompaction (LVNC) has been associated with an increased risk of adverse cardiovascular events, the accurate incidence of cardiovascular morbidity and mortality is unknown. We, therefore, aimed to assess the incidence rate of LVNC-related cardiovascular events.

**Methods:**

We systematically searched observational studies reporting the adverse outcomes related to LVNC. The primary end point was cardiovascular mortality.

**Results:**

We identified 28 eligible studies enrolling 2501 LVNC patients (mean age, 46 years; male/female ratio, 1.7). After a median follow-up of 2.9 years, the pooled event rate for cardiovascular mortality was 1.92 (95% CI, 1.54–2.30) per 100 person-years. LVNC patients had a similar risk of cardiovascular mortality compared with a dilated cardiomyopathy control group (odds ratio, 1.10 [95% CI, 0.18–6.67]). The incidence rates of all-cause mortality, stroke and systemic emboli, heart failure admission, cardiac transplantation, ventricular arrhythmias, and cardiac device implantation were 2.16, 1.54, 3.53, 1.24, 2.17, and 2.66, respectively, per 100 person-years. Meta-regression and subgroup analyses revealed that left ventricular ejection fraction, not the extent of left ventricular trabeculation, had an important influence on the variability of incidence rates. The risks of thromboembolism and ventricular arrhythmias in LVNC patients were similar to dilated cardiomyopathy patients. However, LVNC patients had a higher incidence of heart failure hospitalization than dilated cardiomyopathy patients.

**Conclusions:**

Patients with LVNC carry a similar cardiovascular risk when compared with dilated cardiomyopathy patients. Left ventricular ejection fraction—a conventional indicator of heart failure severity, not the extent of trabeculation—appears to be an important determinant of adverse outcomes in LVNC patients.

**Registration:**

https://www.crd.york.ac.uk/PROSPERO/ Unique identifier: CRD42018096313.

## Abstract 2

Supplemental Digital Content is available in the text.

**Clinical Perspective**

In this large meta-analysis of adult patients with left ventricular noncompaction identified by currently accepted imaging criteria, the incidences of objective cardiovascular outcomes appear comparable to those observed in dilated cardiomyopathy. The frequency of adverse outcomes is mostly driven by left ventricular systolic impairment rather than the burden of trabeculation. The diversity of current imaging diagnostic criteria for left ventricular noncompaction creates significant challenges for accurate phenotyping. Further prospective clinical registries with access to individual-level data are required to standardize the left ventricular noncompaction diagnostic criteria, comorbidities, and outcome measures to fully evaluate the prognostic markers of this poorly understood condition.

**See Editorial by Sharain and Anavekar**

Left ventricular noncompaction (LVNC) cardiomyopathy is characterized by prominent left ventricular (LV) trabeculations, deep intertrabecular recesses communicating with the ventricular cavity, and a thin and compacted epicardial layer. While LVNC is considered a genetic cardiomyopathy by the American Heart Association,^1^ the European Society of Cardiology categorizes it as an unclassified cardiomyopathy.^2^ Multiple pathogeneses of the LVNC phenotype have been proposed: it may be familial (inherited) or nonfamilial (sporadic and proven absent in relatives) and may occur as an isolated disease or in association with genetic diseases and congenital defects.^3^ Nonfamilial and sporadic forms have been described in highly trained athletes,^4^ sickle cell anemia,^5^ and pregnancy.^6^ The genetic basis of familial LVNC is still controversial. Most familial cases of LVNC are associated with mutations in the same genes associated with other types of inherited cardiomyopathies (Figure 1A).^7^

**Figure 1.**
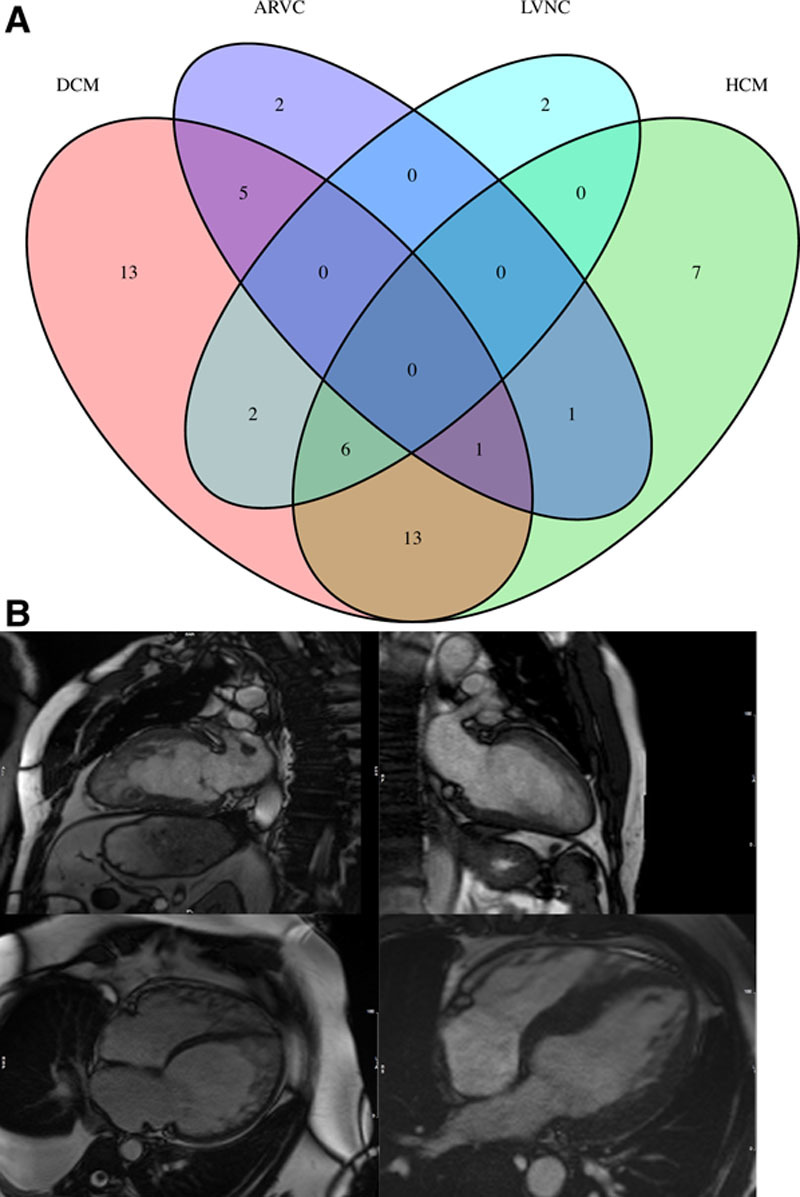
**Genotype and phenotype of LVNC**. **A**, Venn diagram of the number of genes associated with inherited cardiomyopathy; (**B**) cardiovascular magnetic resonance images demonstrating a classic left ventricular noncompaction (LVNC) with a 2-layer appearance of thin compact myocardium and excessive trabeculation (top left), isolated LVNC with normal chamber size and function (top right), mixed dilated cardiomyopathy (DCM) and LVNC with biventricular involvement (bottom left), and hypertrophic cardiomyopathy (HCM) with features of LVNC (bottom right). ARVC indicates arrhythmogenic right ventricular cardiomyopathy.

The diagnosis of LVNC has conventionally been made by imaging the LV and demonstrating the presence of specific criteria based mostly upon the relative thickness of the compacted myocardial wall and the mesh of trabeculated (noncompacted) layer of cardiac muscle using either echocardiography or cardiovascular magnetic resonance imaging (Figure 1B). All current methodologies used to establish a diagnosis have strengths and weaknesses in how they are derived, their ease of use, the time to acquire the relevant images, and their diagnostic accuracy, but there is no evidence to suggest that any particular criteria or imaging modality is superior. However, as image quality and awareness of diagnostic criteria have improved, the LVNC phenotype has emerged as an increasingly recognized finding with the inherent risk of overdiagnosis noted as a significant concern.^8^

The clinical outcomes of LVNC vary widely in the reported literature, which perhaps reflects the underlying diversity of study cohorts. In view of the continued uncertainty, we conducted a systematic review of observational cohort studies to explore the clinical outcomes of patients considered to be affected by LVNC.

## Methods

The data, analytic methods, and study materials can be obtained from the corresponding author for purposes of reproducing the results or replicating the results. Because this is a meta-analysis of aggregate data from the published literature, no informed consent was required. Likewise, because we have not recruited new patients, an institutional review board’s approval was not necessary.

We aimed to explore the adverse outcomes of patients with LVNC through a systematic review of the literature including prospective longitudinal and retrospective observational studies. The complete study protocol was registered on PROSPERO—an international database of prospectively registered systematic reviews—and can be accessed at www.crd.york.ac.uk/PROSPERO/display_record.php?ID=CRD42018096313.

We recognized the challenges associated with meta-analyses of observational studies because of variable study designs and inherent biases. Therefore, we conducted this systematic review following the recommendations by the Meta-Analysis of Observational Studies in Epidemiology group^9^ and the PRISMA (Preferred Reporting Items for Systematic Reviews and Meta-Analyses) guidelines.^10^

### Search Strategy

We searched PubMed and Embase databases, the Cochrane Database of Systematic Reviews, the PROSPERO database (www.crd.york.ac.uk/prospero), and the Clinical Trials Registry (www.clinicaltrials.gov), as well as abstracts from major cardiological societies for potentially relevant articles using a combination of key words related to trabeculation or LVNC and the cardiovascular outcomes for the period from January 1, 1966, to July 3, 2019, without any language restriction. Details of the search terms are provided in the Data Supplement.

### Selection Criteria

Inclusion criteria were (1) patients >18 years old; (2) a diagnosis of LVNC by echocardiographic or cardiovascular magnetic resonance criteria; (3) crude or adjusted event rates of all-cause mortality, cardiovascular mortality, ventricular arrhythmias, sudden cardiac death, heart failure hospitalization, myocardial infarction, stroke, systemic embolic events, new cardiac implantable electronic device, and heart transplantation. Definitions of excessive trabeculation according to cardiac imaging were defined by Petersen et al,^11^ Chin et al,^12^ Jenni et al,^13^ Jacquier et al,^14^ Grothoff et al,^15^ Stacey et al,^16^ Stöllberger et al,^17^ or Captur et al^18^ criteria. We excluded case reports, nonoutcome studies, and reviews.

### Data Extraction

Two authors (F.R., S.D.) performed the screening of titles and abstracts, reviewed the full-text articles, and determined their eligibility. Divergences were solved by consensus or involving the third author (N.A.). We also handsearched the reference list of all eligible articles for additional relevant studies.

We collated study-level covariates and events reported in original publications, using a standardized data extraction form. We translated relevant non-English articles into English. We contacted the authors of studies where clarification of data was required. In studies with overlapping cohorts, we used the data from the most recent study or the study with the largest sample size.

### Quality Assessment

We assessed the individual study-level quality by the Quality in Prognosis Studies tool,^19^ which evaluates 32 key considerations across 6 bias domains: (1) study participation, (2) study attrition, (3) prognostic factor measurement, (4) outcome measurement, (5) study confounding, and (6) statistical analysis and reporting. An overall quality grade (high quality, intermediate quality, and low quality) was assigned to each study after considering all 6 bias domains. Two authors (M.Y.K. and A.A.-B.) independently rated the quality items, and disagreements were resolved by another author (N.A.).

### Outcomes

The primary end point was the incidence of cardiovascular mortality. Secondary end points included incidences of all-cause mortality, stroke and systemic embolic events, heart failure requiring hospitalization, cardiac transplantation, ventricular arrhythmias (ventricular tachycardia or ventricular fibrillation), and cardiac device implantation defined as insertion of implantable cardioverter defibrillator or cardiac synchronization therapy with implantable cardioverter defibrillator.

### Statistical Analysis

Dichotomous variables were reported as percentages, with continuous variables reported as mean±SD or median (interquartile range [IQR]), based on data distribution. For each included study, we calculated an event rate with its 95% CI for every predefined outcome. Event rates were computed as the ratio between the number of events and the person-time in years at risk, to account for the heterogeneity of follow-up duration across different studies. We performed Freeman-Tukey transformation^20^ of the number of events for variance stabilization. We added 0.5 to the count in studies with zero event to achieve numerical stability. For studies reporting the event rates in both LVNC subjects and non-LVNC controls, we calculated odds ratio and 95% CI for each outcome.

We used random-effects models to estimate the summary pooled event rates or odd ratios of prespecified outcomes using the DerSimonian and Laird method. We graphically presented the results in forest plots, with point estimates of the effect size and 95% CI for each study and the combined estimate. The area of squares and diamonds in the forest plots are proportional to each study weight.

We assessed funnel plot asymmetry, which could result from publication bias. We additionally used the Egger regression asymmetry test for end points with asymmetrical funnel plots. We also performed the nonparametric trim-and-fill procedure, which adjusts for funnel plot asymmetry by computing hypothetical missing studies. We formally assessed statistical heterogeneity by a χ^2^ test and quantified it using the inconsistency index (I^2^) statistic, which ranges from 0% to 100% and is defined as the percentage of observed between-trial variability that is due to heterogeneity rather than chance. A lack of homogeneity was considered to be significant with an I^2^ ≥50%. We anticipated a high degree of heterogeneity across individual studies because of the multiplicity of LVNC diagnostic criteria and the variability of inclusion and exclusion criteria used by individual studies. Accordingly, we used random-effects models to account for the between-study variabilities in the effect estimates. To explore the possible reasons of heterogeneity, we performed the following secondary analyses: (1) univariate meta-regression assessing the mediating effect of age, sex (percentage of men), New York Heart Association (NYHA) classification, LV end-diastolic diameter, and LVNC/LV compaction ratio (for thromboembolic end point, we additionally investigated the mediating effects of the percentage of prevalent atrial fibrillation); (2) subgroup analyses according to person-time at risk in years (sample size multiplied by mean follow-up years), presence of moderate-to-severe LV systolic dysfunction (by LV ejection fraction [LVEF] <45%) at the time of recruitment, and overall quality of included studies. We also sought to compare the event rates of the LVNC patients in our study with a recently published meta-analysis of nonischemic dilated cardiomyopathy (DCM) patients,^21^ which reported the incidences of cardiovascular mortality, heart failure hospitalization, and ventricular arrhythmias. We extracted the sample size, absolute number of events, and follow-up duration of individual studies from this DCM meta-analysis to calculate the incidence rate per 100 person-years. The difference in effect estimates between the disease groups and subgroups was assessed with *Z* test. We evaluated the impact of a single study on the overall pooled estimate in meta-analysis by removing one study at a time and recomputing the pooled result; this procedure is known as leave-one-out analysis. Additional details on the statistical tests were outlined in the Data Supplement.

A 2-sided *P*<0.05 was considered statistically significant. We performed all analyses and constructed graphs using the metafor package^22^ in R, version (3.5.0).^23^

## Results

**Figure 2.**
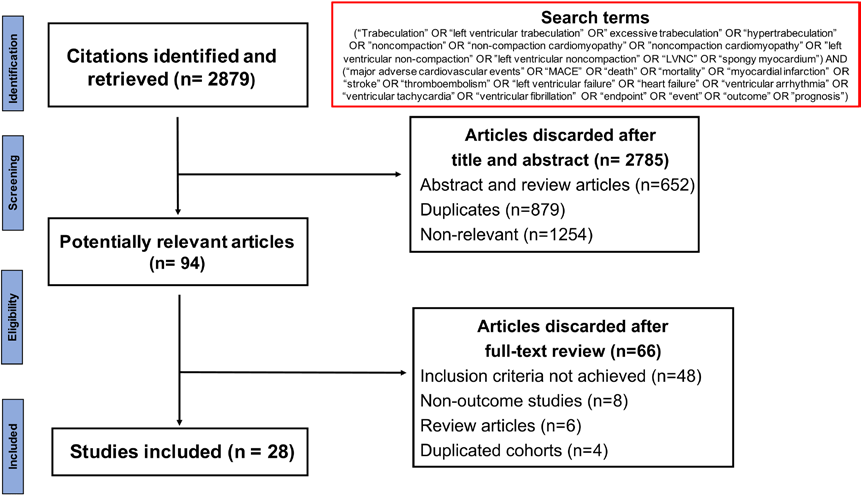
**Flowchart demonstrating the process of study selection.**

Our search strategy yielded 2879 studies, of which 94 full texts were relevant for evaluation (Figure 2). Exclusion of nonrelevant studies, review articles, and studies with duplicated cohorts resulted in 28 publications related to outcomes in LVNC. Searches of the Clinical Trials Registry identified one ongoing study titled “Prognosis of Isolated Left Ventricular Non-Compaction in Adults” in France.

**Figure 3.**
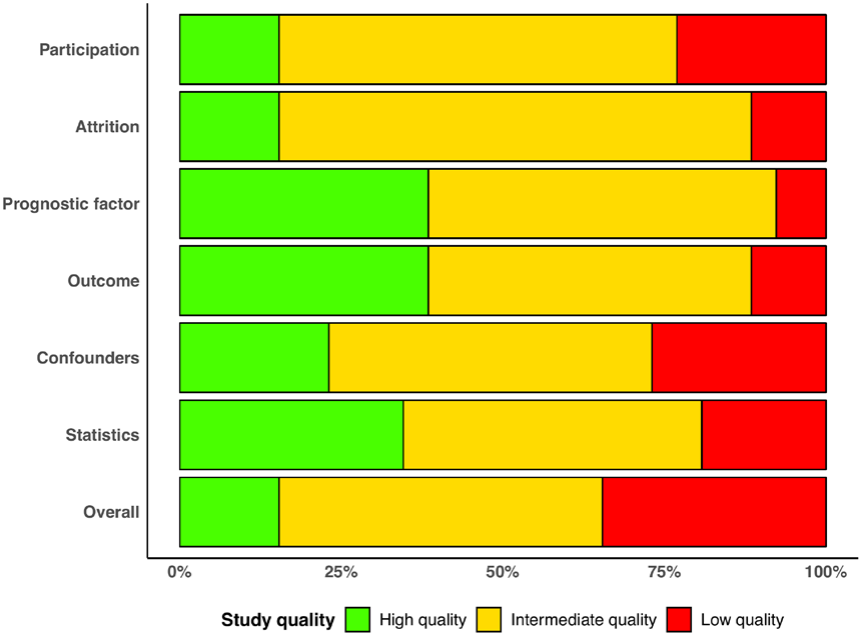
**Distribution of study quality according to Quality in Prognosis Studies tool.**

The final list (28 studies) consisted of 13 prospective and 15 retrospective observational studies. The studies were published between 1997 and 2019. A total of 2501 patients were included (mean±SD age, 46±7 years; male/female ratio, 1.7) with an overall median follow-up of 2.8 (IQR, 2.3–4.1) years. Although the diagnosis of LVNC was based mainly on quantification of excessive trabeculation, the majority of included studies (18 of 28) comprised cohorts with significantly impaired LV systolic function (mean LVEF, <45%). The main characteristics of included studies are presented in Table.^24–51^ Among 28 studies, the distribution of overall study quality was 18%, 50%, and 32% for high, intermediate, and low quality, respectively (Figure 3).

**Table.**
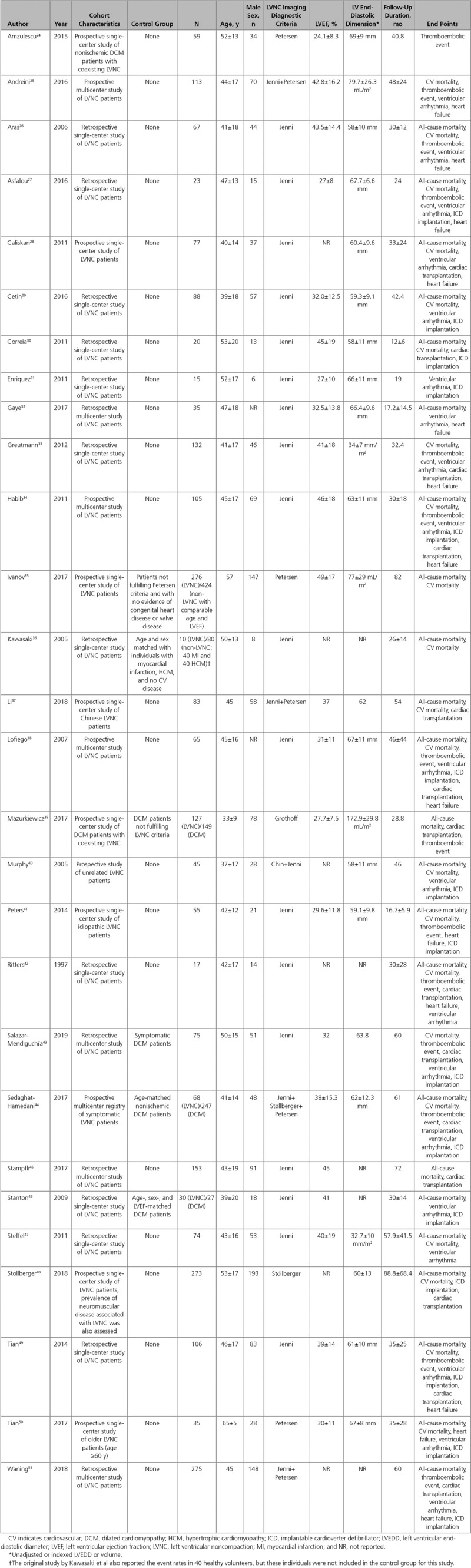
Characteristics of Included Studies

### Primary Outcome

**Figure 4.**
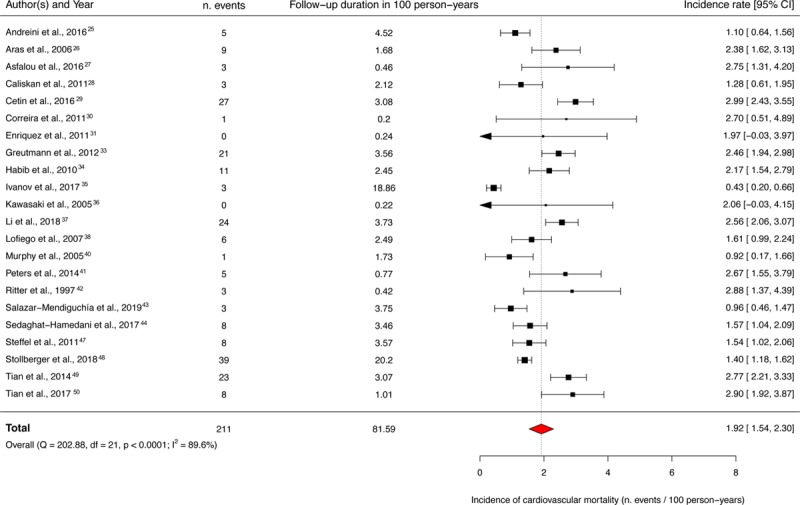
**Forest plot demonstrating the individual and overall incidences of cardiovascular deaths per 100 person-years.** The vertical dotted line indicates the pooled average incidence rate.

**Figure 5.**
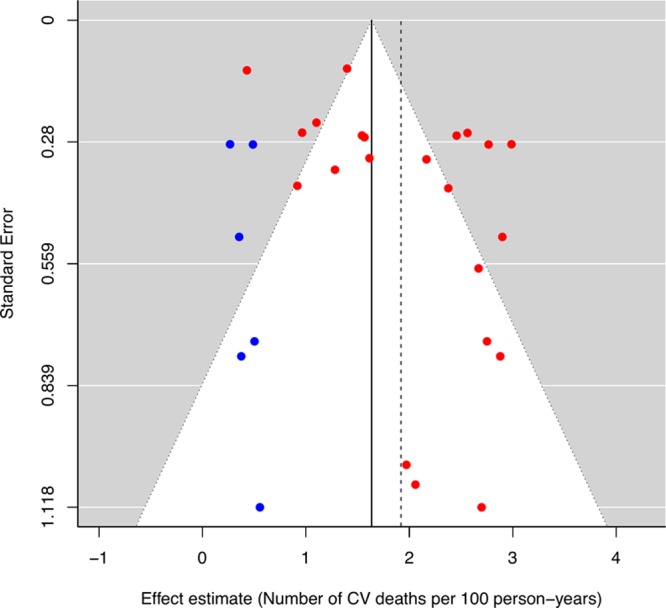
**Funnel plot for cardiovascular (CV) mortality.** The red dots represent the original studies included in the meta-analysis while the blue dots represent the missing studies imputed by the trim-and-fill method. The vertical dashed line indicates the original pooled incidence rates, and the vertical solid line indicates the revised pool incidence rates after inclusion of the imputed missing studies to counter publication bias.

Of 28 included studies, 22 studies provided data on cardiovascular mortality in a total of 1822 patients who were followed up for a median (IQR) duration of 2.9 (2.4–4.4) years. The pooled incidence rate of cardiovascular death was 1.92 (95% CI, 1.54–2.30) per 100 person-years (Figure 4^25–31,33–38,40–44,47–50^). The funnel plot for the primary outcome appeared asymmetrical because of the absence studies in the lower left corner, raising the possibility of publication bias (Egger regression asymmetry test, *P*=0.048). Addition of hypothetical missing studies (n=6) by the trim-and-fill method reduced the pooled cardiovascular mortality rate to 1.64 (95% CI, 1.29–1.98) per 100 person-years (Figure 5).

**Figure 6.**
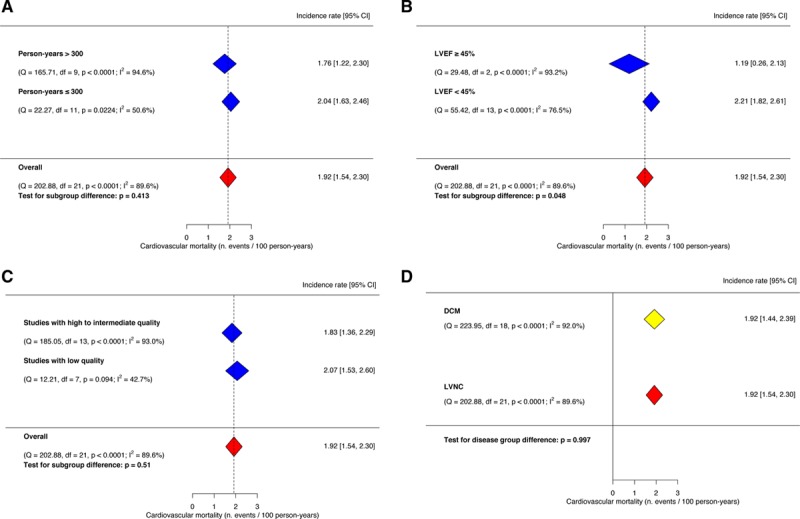
**Subgroup analyses for cardiovascular mortality**. **A** Incidence of cardiovascular mortality in subgroups stratified by person-years >300; (**B**) incidence of cardiovascular mortality in subgroups stratified by left ventricular ejection fraction (LVEF) <45%; (**C**) incidence of cardiovascular mortality in subgroups stratified by high vs low-moderate risk of bias; (**D**) incidence of cardiovascular mortality in left ventricular noncompaction (LVNC) meta-analysis vs external dilated cardiomyopathy (DCM) meta-analysis. The vertical dotted line indicates the pooled average incidence rate.

We observed a substantial between-study heterogeneity (I^2^=89.6%; *P*<0.0001). Therefore, we explored the clinical and statistical sources of heterogeneity by meta-regression and subgroup analyses. The meta-regression analyses investigating the mediating effects of age, proportion of men, proportion of patients with NYHA >2, LV end-diastolic diameter, and LVNC/LV compaction ratio did not identify any significant association. In subgroup analyses, studies enrolling patients with moderate-to-severe LV impairment (LVEF <45%) appeared to have a higher incidence of cardiovascular mortality, compared with studies including patients with mildly impaired or normal LV systolic function (LVEF ≥45%; 2.21 [95% CI, 1.82–2.61] cardiovascular deaths per 100 person-years, I^2^=76.5%, versus 1.19 [95% CI, 0.26–2.13] cardiovascular deaths per 100 person-years, I^2^=93.2%; *P* for subgroup difference, 0.048). There was no significant difference in event rates when stratified by person-time at risk (an amalgamation of sample size and follow-up duration) of >3 years and high/intermediate versus low-quality studies (Figure 6).

The overall estimate of cardiovascular mortality did not change significantly in the leave-one-out sensitivity analysis indicating that no single study had an overwhelming impact on the combined meta-analysis estimate (Figure I in the Data Supplement).

### Secondary Outcomes

#### All-Cause Mortality

Twenty-four studies documented the incidence of all-cause mortality in 2122 patients during a median (IQR) follow-up of 2.6 (2.1–4.0) years. The pooled incidence rate of all-cause mortality was 2.16 (95% CI, 1.90–2.42) per 100 person-years (Figure II in the Data Supplement). The funnel plot and Egger regression asymmetry test suggest possible publication bias (Egger test, *P*=0.006). After addition of 6 hypothetical studies in the trim-and-fill sensitivity analysis, the pooled incidence rate decreased to 1.88 (95% CI, 1.60–2.16) per 100 person-years.

There was a substantial statistical heterogeneity among studies (I^2^=78.1%; *P*<0.0001). In meta-regression analyses, the proportion of male sex and the percentage of individuals with NYHA >2 were positively associated with all-cause mortality. In subgroups stratified by LVEF, studies including patients with moderate-to-severe LV impairment (LVEF <45%) appeared to have a higher incidence of all-cause deaths (*P* for subgroup difference, 0.011). The leave-one-out analysis was consistent with the overall result.

#### Stroke and Systemic Emboli

The event rates of stroke and systemic emboli were reported in 15 studies accounting for 1332 patients with a median (IQR) follow-up of 2.7 (2.4–3.8) years. The pooled incidence rate was 1.54 (95% CI, 1.22–1.86) per 100 person-years (Figure III in the Data Supplement). We did not observe asymmetry in the funnel plot. Similar to the primary outcome, we identified a substantial heterogeneity among studies (I^2^=73.4%; *P*<0.0001). Meta-regression analyses did not reveal any mediating influence of age, sex, NYHA classification, LV end-diastolic diameter or prevalent atrial fibrillation. Stratification by the study quality, LVEF, or person-time at risk did not show significant differences in the events rates between subgroups. The leave-one-out sensitivity analysis showed no evidence of bias introduced by any one study.

#### Heart Failure Hospitalization

Twelve studies (1028 patients; median [IQR] follow-up, 2.5 [2.1–2.9] years) reported the incidence of heart failure hospitalization. The pooled event rate of heart failure hospitalization was 3.53 (95% CI, 2.95–4.11) per 100 person-years (Figure IV in the Data Supplement). The funnel plot did not appear asymmetrical. There was a considerable between-study heterogeneity (I^2^=87.7%; *P*<0.0001). Meta-regression analyses identified a positive association between the proportion of symptomatic heart failure (NYHA >2) at baseline and the incidence of heart failure admission at follow-up (regression coefficient, 0.04 per 1% increase in proportion of cohort with NYHA >2; *P*=0.049). In subgroup analyses, studies with an aggregate person-time at risk >300 years appeared to have a lower incidence rate (2.77 [95% CI, 1.89–3.66], I^2^=92.5%, versus 3.97 [95% CI, 3.34–4.60] per 100 person-years, I^2^=71.6%; *P* for subgroup difference, 0.031). The leave-one-out analysis was consistent with the overall pooled estimate.

#### Heart Transplantation

Data on cardiac transplantation rate were available in 14 studies (1576 patients; median [IQR] follow-up, 2.8 [2.4–4.9] years). The overall pooled event rate of heart transplantation was 1.24 (95% CI, 0.98–1.50) per 100 person-years (Figure V in the Data Supplement). The funnel plot showed sparsely distributed studies with evidence of asymmetry (Egger *P*<0.0001). After addition of 1 hypothetical study in the trim-and-fill sensitivity analysis, the pooled incidence rate decreased minimally to 1.22 (95% CI, 0.96–1.48) per 100 person-years. The statistical heterogeneity of studies reporting heart transplantation outcome was substantial (I^2^=71.6%; *P*<0.0001). Meta-regression analyses did not reveal any mediating effect of the selected covariates. We again found a lower incidence of heart transplantation in the subgroup with an aggregate person-time at risk >300 years (1.04 [95% CI, 0.81–1.26], I^2^=60.3%, versus 1.79 [95% CI, 1.31–2.27] per 100 person-years, I^2^=35.1%; *P* for subgroup difference, 0.005). No undue influence from any single study was detected in the leave-one-out analysis.

#### Ventricular Arrhythmias

Nineteen studies with a total sample size of 1445 (median [IQR] follow-up of 2.8 [2.4–3.8] years) documented the incidence of ventricular arrhythmias. The calculated pooled event rate was 2.17 (95% CI, 1.78–2.56) per 100 person-years (Figure VI in the Data Supplement). There was no convincing evidence of publication bias in the funnel plot (Egger *P*=0.05). We identified a substantial heterogeneity among studies (I^2^=84.4%; *P*<0.0001). Meta-regression analyses did not find any significant association with covariates, but the subgroup with moderate-severe LV impairment (LVEF <45%) appeared to have a higher incidence of ventricular arrhythmias (2.30 [95% CI, 1.72–2.88], I^2^=88.4%, versus 1.60 [95% CI, 1.23–1.97] per 100 person-years, I^2^=0%; *P* for subgroup difference, 0.047). The leave-one-out analysis did not show any significant deviation from the overall pooled result.

#### Cardiac Device Implantation

The incidence of cardiac device implantation was recorded in 15 studies (1278 patients; median [IQR] follow-up of 2.9 [2.0–3.8] years). The pooled incidence rate was 2.66 (95% CI, 1.93–3.39) per 100 person-years (Figure VII in the Data Supplement). The funnel plot appeared sparse but symmetrical. A considerable between-study heterogeneity was present (I^2^=95.3%; *P*<0.0001). The meta-regression analyses identified a negative association between the proportion of male sex and the incidence of cardiac device implantation (regression coefficient, −0.06 per 1% increase in male sex proportion; *P*=0.04). Stratified analyses did not find any significant subgroup difference although the inconsistency index (I^2^) appeared much lower in some subgroups. We did not find any indication of bias in the leave-one-out analysis.

#### Comparison With DCM

**Figure 7.**
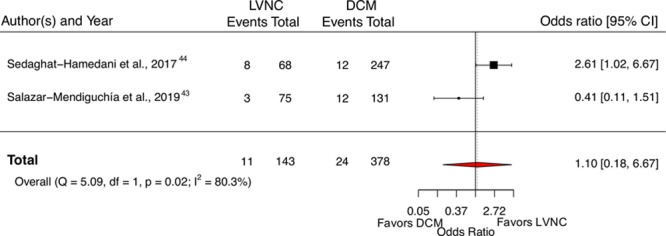
**Forest plot of cardiovascular mortality in left ventricular noncompaction (LVNC) patients compared with dilated cardiomyopathy (DCM) controls.** The vertical dotted line represents the pooled odds ratio.

Two studies of 22 reported the incidence of cardiovascular death in a comparable group of DCM patients. Overall, the LVNC patients did not have significantly higher cardiovascular mortality than the DCM group (pooled odds ratio, 1.10 [95% CI, 0.18–6.67]; Figure 7^43,44^). The pooled event rate of cardiovascular death in a previously published meta-analysis of DCM patients^21^ (19 studies enrolling 2466 individuals) was comparable to the pooled event rate observed in our study (DCM, 1.92 [95% CI, 1.44–2.39] cardiovascular deaths per 100 person-years versus LVNC, 1.92 [95% CI, 1.54–2.30] cardiovascular deaths per 100 person-years; Figure 6D). Two studies of 24 provided all-cause mortality data in a DCM control group. In comparison with the DCM group, patients with LVNC did not have significantly higher mortality (pooled odds ratio, 0.67 [95% CI, 0.28–1.59]). When compared with an external previously published DCM meta-analysis,^21^ the heart failure hospitalization rate in our study was significantly higher (3.53 versus 2.37 per 100 person-years; *P*=0.003). There was no significant difference in the incidence rate of ventricular arrhythmias between our study and the previous DCM meta-analysis^21^ (2.17 for LVNC versus 2.14 for DCM per 100 person-years; *P*=0.93).

## Discussion

In this meta-analysis investigating the prognosis of a large population of adult LVNC patients classified according to contemporary imaging criteria, we identified the following key findings: (1) the overall incidence rates of cardiovascular mortality, all-cause mortality, stroke and systemic emboli, heart failure admission, cardiac transplantation, ventricular arrhythmias, and cardiac device implantation were 1.92, 2.16, 1.54, 3.53, 1.24, 2.17, and 2.66, respectively, per 100 person-years, at an intermediate-term follow-up; (2) the incidence of cardiovascular or all-cause mortality in LVNC patients was similar to DCM controls; (3) the high level of statistical heterogeneity was partly explained by the variability in clinical characteristics (LVEF in particular) and study characteristics such as sample size/study duration; (4) the incidence rate of ventricular arrhythmias was comparable to DCM patients, but heart failure admission rate was higher in LVNC patients.

By investigating the prognosis of real-world patients with excessive trabeculations meeting the imaging diagnostic criteria for LVNC, we aimed to provide much needed information on the natural course of this controversial disease entity. The findings from our study can be regarded as a foundation for further discussion regarding the medical implications of an increasingly recognized imaging finding and also highlight important heterogeneity among available published studies.

The incidence rates of cardiovascular and all-cause mortality, arguably, 2 more reliable and objective outcomes, estimated to be 1.92 and 2.14 per 100 person-years, respectively, in our meta-analysis, are 25- and 5-fold higher than the event rates in a general population (0.08 and 0.41 per 100 person-years for cardiovascular and all-cause mortality, respectively, in 45- to 54-year age group in a North American population).^52^ Therefore, a diagnosis of LVNC by current clinical and imaging criteria appears to portend a heightened mortality risk despite a significant diversity of patient population in the individual studies. Nonetheless, when compared with nonischemic DCM patients, LVNC patients carry a similar risk of death from cardiovascular causes. We also observed elevated incidences of cardiovascular morbidities in LVNC patients with 2 most frequent complications being heart failure hospitalization and cardiac device implantation. The heart failure–related hospital admission rate in our meta-analysis was higher than the pooled incidence observed in a comparable DCM meta-analysis (3.52 versus 2.37 per 100 person-years). This finding should be interpreted with caution in view of variability in definition of heart failure decompensation and lack of data on the rigor of heart failure treatment.

There is a notion of an increased risk of systemic thromboembolism attributable to the sluggish blood flow in the deep intertrabecular recesses in LVNC patients. However, no solid evidence is available to support this hypothesis. Indeed, in our study, the incidence rate of stroke and systemic emboli was 1.54 per 100 person-years, which is either lower than or comparable to the event rates reported in (1) V-HeFT trials in patients with systolic heart failure (2.1–2.7 per 100 person-years), (2) patients with ischemic cardiomyopathy (1.5 per 100 person-years) in SAVE trial,^53^ and (3) DCM patients (3.5 per 100 person-years).^54,55^

It is important to consider the incidence rates reported in this meta-analysis in the context of cohort characteristics where 18 of 28 included studies recruited individuals with significant LV systolic impairment. Subgroup analysis stratified by LVEF demonstrated a significant reduction in the number of cardiovascular deaths in the absence of moderate-to-severe LV systolic dysfunction. Therefore, the risk of achieving the end point may, in part, be contingent on the development of LV dysfunction. Although the risk to individuals with excessive trabeculations in an unselected and otherwise healthy population is beyond the scope of this study, a previous population study of ≈3000 asymptomatic individuals did not find any association between the degree of trabeculation and the decline in LV function or incident cardiovascular events over a course of ≈10 years.^56^

As anticipated, we observed a high degree of statistical heterogeneity among included studies that can be partially explained by the differences in cohort characteristics and study quality. In our quality assessment by Quality in Prognosis Studies criteria, the 2 most commonly affected bias domains were study participation (ie, selection bias) and treatment of confounders, reflecting the challenges associated with the observational studies reporting a relatively rare condition. The subsequent meta-regression and subgroup analyses revealed that severity of LV impairment measured by LVEF had an important influence on the variability of incidence rates reported in individual studies. Equivalently, smaller studies with short follow-up duration tended to report higher incidence rates of secondary outcomes. The indicator of between-study variability (I^2^ index) was noticeably lower in the subgroup analysis, which further supports the importance of well-defined inclusion and diagnostic criteria.

All imaging diagnostic criteria for LVNC consider the presence of excessive trabeculation as a cardinal signature of disease. There is a degree of confusion and uncertainty in assigning the disease status because of not-so-infrequent finding of increased trabeculation in otherwise healthy individuals and those with primary DCM or hypertrophic cardiomyopathy. Recent evidence suggests that the extent of trabeculation in asymptomatic low-risk population, LVNC, and DCM patients does not determine prognosis.^24,25,56^ In this respect, the lack of mediating influence by the LVNC/LV compaction ratio on clinical outcomes in our study is concordant with the existing evidence in literature. A recently published meta-analysis of 4 cardiovascular magnetic resonance studies enrolling LVNC patients reported that in the absence of late gadolinium enhancement and LV systolic dysfunction, no hard cardiac event was observed.^57^ Therefore, our study, together with mounting evidence from existing literature, underscores the important prognostic role of focal myocardial injury and functional impairment, rather than the morphological appearance of LVNC. Indeed, it is notable that the conventional diagnostic criteria for LVNC have relied principally on ratio measurement and have not included other structural, functional, clinical, or familial parameters.

In this study, we systematically reviewed and performed the meta-analysis of the incidence of important cardiovascular outcomes in a large population of real-world LVNC patients. We attempted to synthesize the results in a robust manner giving due consideration to address potential biases where possible. However, we acknowledge several limitations associated with our study. First, the pooled analysis relied on observational, mostly single-center, cohort studies with variable methodological quality as highlighted in the bias assessment, inclusion criteria, and definitions of LVNC. Second, our study only focused on the adult population mostly free from congenital heart disease; thus, the insights obtained from this work cannot be extended to pediatric LVNC or patients with coexisting congenital heart disease. Third, comparison of the rates of incident cardiovascular events between LVNC and hypertrophic cardiomyopathy was not performed and should be investigated in a future study. Fourth, meta-regression analyses were limited to the studies without missing covariate information, hence, may be underpowered. Fifth, only a few studies reported the incidence rates in a comparable DCM cohort. Thus, the precision of pooled odds ratio and the level of evidence are weaker. Finally, the incidence rates of adverse events observed in this study only hold true for an intermediate follow-up duration, and the long-term consequences of LVNC remain to be elucidated.

An expert group consensus approach to harmonize the diagnostic criteria, risk factors, and end points is urgently needed to develop a more standardized assessment of LVNC. Future studies including prospective registries should address long-term prognosis and could also investigate additional prognostic information provided by fractal analysis, T1 mapping, and genotype over current LVNC/LV compaction ratio, systolic function, and tissue characterization by late gadolinium enhancement.

## Conclusions

Patients with LVNC have similar risks of cardiovascular mortality, all-cause mortality, thromboembolic complications, and ventricular arrhythmias in comparison with DCM patients. The finding of increased incidence of heart failure hospitalization in isolation should be interpreted with caution and investigated in future, well-designed studies. Traditional indicators of cardiac disease severity such as low LVEF, not the burden of trabeculation, appear to be associated with worse outcomes.

## Acknowledgments

This work was part of the portfolio of translational research of the NIHR Biomedical Research Centre at Barts and The London School of Medicine and Dentistry.

## Sources of Funding

Dr Aung is supported by a Wellcome Trust Research Training Fellowship (203553/Z/Z). Drs Petersen and Aung acknowledge the support through the Barts Biomedical Research Centre, which is funded by the National Institute for Health Research.

## Disclosures

Dr Petersen provides consultancy to Circle Cardiovascular Imaging, Inc, Calgary, Canada. The authors report no conflicts.

## Supplementary Material

## References

[1] Maron BJ, Towbin JA, Thiene G, Antzelevitch C, Corrado D, Arnett D, Moss AJ, Seidman CE, Young JB, American Heart Association (2006). Contemporary definitions and classification of the cardiomyopathies: an American Heart Association Scientific Statement from the Council on Clinical Cardiology, Heart Failure and Transplantation Committee; Quality of Care and Outcomes Research and Functional Genomics and Translational Biology Interdisciplinary Working Groups; and Council on Epidemiology and Prevention.. Circulation.

[2] Elliott P, Andersson B, Arbustini E, Bilinska Z, Cecchi F, Charron P, Dubourg O, Kühl U, Maisch B, McKenna WJ (2008). Classification of the cardiomyopathies: a position statement from the European Society Of Cardiology Working Group on Myocardial and Pericardial Diseases.. Eur Heart J.

[3] Arbustini E, Weidemann F, Hall JL (2014). Left Ventricular Noncompaction: A Distinct Cardiomyopathy or a Trait Shared by Different Cardiac Diseases?. J Am Coll Cardiol.

[4] Gati S, Chandra N, Bennett RL, Reed M, Kervio G, Panoulas VF, Ghani S, Sheikh N, Zaidi A, Wilson M (2013). Increased left ventricular trabeculation in highly trained athletes: do we need more stringent criteria for the diagnosis of left ventricular non-compaction in athletes?. Heart Br Card Soc.

[5] Gati S, Papadakis M, Van Niekerk N, Reed M, Yeghen T, Sharma S (2013). Increased left ventricular trabeculation in individuals with sickle cell anaemia: physiology or pathology?. Int J Cardiol.

[6] Gati S, Papadakis M, Papamichael ND, Zaidi A, Sheikh N, Reed M, Sharma R, Thilaganathan B, Sharma S (2014). Reversible de novo left ventricular trabeculations in pregnant women: implications for the diagnosis of left ventricular noncompaction in low-risk populations.. Circulation.

[7] Teekakirikul P, Kelly MA, Rehm HL, Lakdawala NK, Funke BH (2013). Inherited Cardiomyopathies: Molecular Genetics and Clinical Genetic Testing in the Postgenomic Era.. J Mol Diagn.

[8] Ross SB, Jones K, Blanch B, Puranik R, McGeechan K, Barratt A, Semsarian C (2019). A systematic review and meta-analysis of the prevalence of left ventricular non-compaction in adults.. Eur Heart J.

[9] Stroup DF, Berlin JA, Morton SC, Olkin I, Williamson GD, Rennie D, Moher D, Becker BJ, Sipe TA, Thacker SB (2000). Meta-analysis of Observational Studies in Epidemiology: A Proposal for Reporting.. JAMA.

[10] Moher D, Liberati A, Tetzlaff J, Altman DG, Group TP (2009). Preferred Reporting Items for Systematic Reviews and Meta-Analyses: The PRISMA Statement.. PLOS Med.

[11] Petersen SE, Selvanayagam JB, Wiesmann F, Robson MD, Francis JM, Anderson RH, Watkins H, Neubauer S (2005). Left Ventricular Non-Compaction: Insights From Cardiovascular Magnetic Resonance Imaging.. J Am Coll Cardiol.

[12] Chin TK, Perloff JK, Williams RG, Jue K, Mohrmann R (1990). Isolated noncompaction of left ventricular myocardium. A study of eight cases.. Circulation.

[13] Jenni R, Oechslin E, Schneider J, Jost C, Kaufmann P (2001). Echocardiographic and pathoanatomical characteristics of isolated left ventricular non-compaction: a step towards classification as a distinct cardiomyopathy.. Heart.

[14] Jacquier A, Thuny F, Jop B, Giorgi R, Cohen F, Gaubert J-Y, Vidal V, Bartoli JM, Habib G, Moulin G (2010). Measurement of trabeculated left ventricular mass using cardiac magnetic resonance imaging in the diagnosis of left ventricular non-compaction.. Eur Heart J.

[15] Grothoff M, Pachowsky M, Hoffmann J, Posch M, Klaassen S, Lehmkuhl L, Gutberlet M (2012). Value of cardiovascular MR in diagnosing left ventricular non-compaction cardiomyopathy and in discriminating between other cardiomyopathies.. Eur Radiol.

[16] Stacey RB, Andersen MM, St Clair M, Hundley WG, Thohan V (2013). Comparison of systolic and diastolic criteria for isolated LV noncompaction in CMR.. JACC Cardiovasc Imaging.

[17] Stöllberger C, Gerecke B, Finsterer J, Engberding R (2013). Refinement of echocardiographic criteria for left ventricular noncompaction.. Int J Cardiol.

[18] Captur G, Muthurangu V, Cook C, Flett AS, Wilson R, Barison A, Sado DM, Anderson S, McKenna WJ, Mohun TJ (2013). Quantification of left ventricular trabeculae using fractal analysis.. J Cardiovasc Magn Reson.

[19] Hayden JA, van der Windt DA, Cartwright JL, Côté P, Bombardier C (2013). Assessing bias in studies of prognostic factors.. Ann Intern Med.

[20] Freeman MF, Tukey JW (1950). Transformations Related to the Angular and the Square Root.. Ann Math Stat.

[21] Becker MAJ, Cornel JH, van de Ven PM, van Rossum AC, Allaart CP, Germans T (2018). The Prognostic Value of Late Gadolinium-Enhanced Cardiac Magnetic Resonance Imaging in Nonischemic Dilated Cardiomyopathy: A Review and Meta-Analysis.. JACC Cardiovasc Imaging.

[22] Viechtbauer W (2010). Conducting meta-analyses in R with the metafor package.. J Stat Softw.

[23] R Core Team (2016). R: A Language and Environment for Statistical Computing [Internet].

[24] Amzulescu M-S, Rousseau MF, Ahn SA, Boileau L, de Meester de Ravenstein C, Vancraeynest D, Pasquet A, Vanoverschelde JL, Pouleur A-C, Gerber BL (2015). Prognostic Impact of Hypertrabeculation and Noncompaction Phenotype in Dilated Cardiomyopathy: A CMR Study.. JACC Cardiovasc Imaging.

[25] Andreini D, Pontone G, Bogaert J, Roghi A, Barison A, Schwitter J, Mushtaq S, Vovas G, Sormani P, Aquaro GD (2016). Long-Term Prognostic Value of Cardiac Magnetic Resonance in Left Ventricle Noncompaction: A Prospective Multicenter Study.. J Am Coll Cardiol.

[26] Aras D, Tufekcioglu O, Ergun K, Ozeke O, Yildiz A, Topaloglu S, Deveci B, Sahin O, Kisacik HL, Korkmaz S (2006). Clinical features of isolated ventricular noncompaction in adults long-term clinical course, echocardiographic properties, and predictors of left ventricular failure.. J Card Fail.

[27] Asfalou I, Boulaamayl S, Raissouni M, Mouine N, Sabry M, Kheyi J, Doghmi N, Benyass A (2017). Left ventricular noncompaction-A rare form of cardiomyopathy: Revelation modes and predictors of mortality in adults through 23 cases.. J Saudi Heart Assoc.

[28] Caliskan K, Szili-Torok T, Theuns DAMJ, Kardos A, Geleijnse ML, Balk AHMM, van Domburg RT, Jordaens L, Simoons ML (2011). Indications and outcome of implantable cardioverter-defibrillators for primary and secondary prophylaxis in patients with noncompaction cardiomyopathy.. J Cardiovasc Electrophysiol.

[29] Cetin MS, Ozcan Cetin EH, Canpolat U, Cay S, Topaloglu S, Temizhan A, Aydogdu S (2016). Usefulness of Fragmented QRS Complex to Predict Arrhythmic Events and Cardiovascular Mortality in Patients With Noncompaction Cardiomyopathy.. Am J Cardiol.

[30] Correia E, Rodrigues B, Santos L, Faria R, Ferreira P, Gama P, Nascimento C, Dionisio O, Cabral C, Santos O (2011). Noncompaction of the ventricular myocardium: characterization and follow-up of an affected population.. Rev Port Cardiol Orgao Of Soc Port Cardiol Port J Cardiol Off J Port Soc Cardiol.

[31] Enríquez R A, Baeza V R, Gabrielli N L, Córdova A S, Castro G P (2011). Non compaction cardiomyopathy: a series of 15 cases.. Rev Médica Chile.

[32] Gaye ND, Ngaïdé AA, Bah MB, Babaka K, Mbaye A, Abdoul K (2017). Non-compaction of left ventricular myocardium in sub-Saharan African adults.. Heart Asia.

[33] Greutmann M, Mah ML, Silversides CK, Klaassen S, Attenhofer Jost CH, Jenni R, Oechslin EN (2012). Predictors of adverse outcome in adolescents and adults with isolated left ventricular noncompaction.. Am J Cardiol.

[34] Habib G, Charron P, Eicher J-C, Giorgi R, Donal E, Laperche T, Boulmier D, Pascal C, Logeart D, Jondeau G (2011). Isolated left ventricular non-compaction in adults: clinical and echocardiographic features in 105 patients. Results from a French registry.. Eur J Heart Fail.

[35] Ivanov Alexander, Dabiesingh Devindra S., Bhumireddy Geetha P., Mohamed Ambreen, Asfour Ahmed, Briggs William M., Ho Jean, Khan Saadat A., Grossman Alexandra, Klem Igor (2017). Prevalence and Prognostic Significance of Left Ventricular Noncompaction in Patients Referred for Cardiac Magnetic Resonance Imaging.. Circ Cardiovasc Imaging.

[36] Kawasaki T, Azuma A, Taniguchi T, Asada S, Kamitani T, Kawasaki S, Matsubara H, Sugihara H (2005). Heart rate variability in adult patients with isolated left ventricular noncompaction.. Int J Cardiol.

[37] Li Shijie, Zhang Ce, Liu Nana, Bai Hui, Hou Cuihong, Wang Jizheng, Song Lei, Pu Jielin (2018). Genotype-Positive Status Is Associated With Poor Prognoses in Patients With Left Ventricular Noncompaction Cardiomyopathy.. J Am Heart Assoc.

[38] Lofiego C, Biagini E, Pasquale F, Ferlito M, Rocchi G, Perugini E, Bacchi-Reggiani L, Boriani G, Leone O, Caliskan K (2007). Wide spectrum of presentation and variable outcomes of isolated left ventricular non-compaction.. Heart Br Card Soc.

[39] Mazurkiewicz Ł, Petryka J, Śpiewak M, Miłosz-Wieczorek B, Małek ŁA, Jasińska A, Jarmus E, Marczak M, Misko J, Grzybowski J (2017). Clinical and prognostic relevancy of left ventricular trabeculation assessed by cardiac magnetic resonance in patients with dilated cardiomyopathy.. Kardiologia Pol Pol Heart J.

[40] Murphy RT, Thaman R, Blanes JG, Ward D, Sevdalis E, Papra E, Kiotsekoglou A, Kiotsekolglou A, Tome MT, Pellerin D (2005). Natural history and familial characteristics of isolated left ventricular non-compaction.. Eur Heart J.

[41] Peters F, Khandheria BK, Botha F, Libhaber E, Matioda H, Dos Santos C, Govender S, Meel R, Essop MR (2014). Clinical outcomes in patients with isolated left ventricular noncompaction and heart failure.. J Card Fail.

[42] Ritter M, Oechslin E, Sütsch G, Attenhofer C, Schneider J, Jenni R (1997). Isolated noncompaction of the myocardium in adults.. Mayo Clin Proc.

[43] Salazar-Mendiguchía J, González-Costello J, Oliveras T, Gual F, Lupón J, Manito N (2019). Long-term Follow-up of Symptomatic Adult Patients With Noncompaction Cardiomyopathy.. Rev Esp Cardiol Engl Ed.

[44] Sedaghat-Hamedani F, Haas J, Zhu F, Geier C, Kayvanpour E, Liss M, Lai A, Frese K, Pribe-Wolferts R, Amr A (2017). Clinical genetics and outcome of left ventricular non-compaction cardiomyopathy.. Eur Heart J.

[45] Stämpfli SF, Erhart L, Hagenbuch N, Stähli BE, Gruner C, Greutmann M, Niemann M, Kaufmann BA, Jenni R, Held L (2017). Prognostic power of NT-proBNP in left ventricular non-compaction cardiomyopathy.. Int J Cardiol.

[46] Stanton C, Bruce C, Connolly H, Brady P, Syed I, Hodge D, Asirvatham S, Friedman P (2009). Isolated left ventricular noncompaction syndrome.. Am J Cardiol.

[47] Steffel J, Hürlimann D, Namdar M, Despotovic D, Kobza R, Wolber T, Holzmeister J, Haegeli L, Brunckhorst C, Lüscher TF (2011). Long-term follow-up of patients with isolated left ventricular noncompaction: role of electrocardiography in predicting poor outcome.. Circ J Off J Jpn Circ Soc.

[48] Stöllberger C, Wegner C, Finsterer J (2019). Left ventricular hypertrabeculation/noncompaction, cardiac phenotype, and neuromuscular disorders.. Herz.

[49] Tian T, Liu Y, Gao L, Wang J, Sun K, Zou Y, Wang L, Zhang L, Li Y, Xiao Y (2014). Isolated left ventricular noncompaction: clinical profile and prognosis in 106 adult patients.. Heart Vessels.

[50] Tian T, Yang K-Q, Mao Y, Zhou L-L, Wang L-P, Xiao Y, Yang Y-K, Zhang Y, Meng X, Zhou X-L (2017). Left Ventricular Noncompaction in Older Patients.. Am J Med Sci.

[51] van Waning JI, Caliskan K, Hoedemaekers YM, van Spaendonck-Zwarts KY, Baas AF, Boekholdt SM, van Melle JP, Teske AJ, Asselbergs FW, Backx APCM (2018). Genetics, Clinical Features, and Long-Term Outcome of Noncompaction Cardiomyopathy.. J Am Coll Cardiol.

[52] Heron M Deaths: Leading Causes for 2016 [Internet].. https://www.cdc.gov/nchs/data/nvsr/nvsr67/nvsr67_06.pdf.

[53] Loh E, Sutton MStJ, Wun C-CC, Rouleau JL, Flaker GC, Gottlieb SS, Lamas GA, Moyé LA, Goldhaber SZ, Pfeffer MA (1997). Ventricular Dysfunction and the Risk of Stroke after Myocardial Infarction.. N Engl J Med.

[54] Fuster V, Gersh BJ, Giuliani ER, Tajik AJ, Brandenburg RO, Frye RL (1981). The natural history of idiopathic dilated cardiomyopathy.. Am J Cardiol.

[55] Koniaris LS, Goldhaber SZ (1998). Anticoagulation in Dilated Cardiomyopathy.. J Am Coll Cardiol.

[56] Zemrak F, Ahlman MA, Captur G, Mohiddin SA, Kawel-Boehm N, Prince MR, Moon JC, Hundley WG, Lima JAC, Bluemke DA (2014). The relationship of left ventricular trabeculation to ventricular function and structure over a 9.5-year follow-up: the MESA study.. J Am Coll Cardiol.

[57] Grigoratos C, Barison A, Ivanov A, Andreini D, Amzulescu M-S, Mazurkiewicz L, De Luca A, Grzybowski J, Masci PG, Marczak M (2019). Meta-Analysis of the Prognostic Role of Late Gadolinium Enhancement and Global Systolic Impairment in Left Ventricular Noncompaction.. JACC Cardiovasc Imaging.

