# Supplementary material for: Prognostic Significance of Left Ventricular Noncompaction: Systematic Review and Meta-Analysis of Observational Studies
Source: Circ Cardiovasc Imaging. 2020 Jan 21;13(1):e009712. doi: 10.1161/CIRCIMAGING.119.009712 (PMC7012350; doi:10.1161/CIRCIMAGING.119.009712)
Supplement: Supplementary file 1 [file hci-13-e009712-s001.docx]

**SUPPLEMENTAL MATERIAL**

**Search terms**

The following search terms were used to query the electronic databases including Pubmed and Embase: (“Trabeculation” OR “left ventricular trabeculation” OR “excessive trabeculation” OR “hypertrabeculation” OR “non-compaction cardiomyopathy” OR “noncompaction” OR “left ventricular non-compaction” OR “left ventricular noncompaction” OR “LVNC” OR “spongy myocardium”) AND (“major adverse cardiovascular events” OR “MACE” OR “death” OR “mortality” OR “myocardial infarction” OR “stroke” OR “thromboembolism” OR “left ventricular failure” OR “heart failure” OR “ventricular arrhythmia” OR “ventricular tachycardia” OR “ventricular fibrillation” OR “endpoint” OR “event” OR “outcome” OR “prognosis”).

**Statistical Methods**

Egger’s regression test examines the funnel plot asymmetry by regressing the standard normal deviate of each study (effect size divided by standard error) against its precision (1/standard error) (1). In the presence of significant funnel plot asymmetry, the regression line will not run through the origin, thus, the intercept 0 provides a measure of asymmetry – the larger its deviation from zero, the more pronounced the asymmetry. The trim and fill method (2) is a non-parametric data augmentation technique used to estimate the number of studies missing from a meta-analysis due to the suppression of the most extreme results on one side of the funnel plot. The algorithm augments the observed data so that the funnel plot is more symmetric and recomputes the summary estimate based on the complete data. This method should be regarded as a sensitivity analysis of the potential effect that missing studies (assumed due to publication bias) may have had on the observed result.

**Supplemental FIGURES**

**Supplemental Figure 1.** Leave-one-out analysis of the pooled incidence rate for cardiovascular mortality

The vertical dashed line indicates the final pooled incidence rate per 100 person-years. Y-axis shows the study which has been excluded from the pooled analysis. Stepwise exclusion of individual studies did not alter the overall pooled estimate.

**Supplemental Figure 2.** Forest plot demonstrating individual and overall incidences of all-cause deaths per 100 person-years

The vertical dotted line indicates the pooled average incidence rate.

**Supplemental Figure 3.** Forest plot demonstrating individual and overall incidences of stroke and systemic emboli per 100 person-years

The vertical dotted line indicates the pooled average incidence rate.

**Supplemental Figure 4.** Forest plot demonstrating individual and overall incidences of heart failure hospitalization per 100 person-years

The vertical dotted line indicates the pooled average incidence rate.

**Supplemental Figure 5.** Forest plot demonstrating individual and overall incidences of cardiac transplantation per 100 person-years

The vertical dotted line indicates the pooled average incidence rate.

**Supplemental Figure 6.** Forest plot demonstrating individual and overall incidences of ventricular arrhythmias per 100 person-years

The vertical dotted line indicates the pooled average incidence rate.

**Supplemental Figure 7.** Forest plot demonstrating individual and overall incidences of cardiac device implantation per 100 person-years

The vertical dotted line indicates the pooled average incidence rate.

**References**

1. Egger M, Davey Smith G, Schneider M, Minder C. Bias in meta-analysis detected by a simple, graphical test. BMJ. 1997 Sep 13;315(7109):629–34.

2. Duval S, Tweedie R. A Nonparametric “Trim and Fill” Method of Accounting for Publication Bias in Meta-Analysis. Journal of the American Statistical Association. 2000 Mar;95(449):89–98.

3. Andreini D, Pontone G, Bogaert J, Roghi A, Barison A, Schwitter J, et al. Long-Term Prognostic Value of Cardiac Magnetic Resonance in Left Ventricle Noncompaction: A Prospective Multicenter Study. J Am Coll Cardiol. 2016 Nov 15;68(20):2166–81.

4. Aras D, Tufekcioglu O, Ergun K, Ozeke O, Yildiz A, Topaloglu S, et al. Clinical features of isolated ventricular noncompaction in adults long-term clinical course, echocardiographic properties, and predictors of left ventricular failure. J Card Fail. 2006 Dec;12(9):726–33.

5. Asfalou I, Boulaamayl S, Raissouni M, Mouine N, Sabry M, Kheyi J, et al. Left ventricular noncompaction-A rare form of cardiomyopathy: Revelation modes and predictors of mortality in adults through 23 cases. J Saudi Heart Assoc. 2017 Apr;29(2):102–9.

6. Caliskan K, Szili-Torok T, Theuns DAMJ, Kardos A, Geleijnse ML, Balk AHMM, et al. Indications and outcome of implantable cardioverter-defibrillators for primary and secondary prophylaxis in patients with noncompaction cardiomyopathy. J Cardiovasc Electrophysiol. 2011 Aug;22(8):898–904.

7. Cetin MS, Ozcan Cetin EH, Canpolat U, Cay S, Topaloglu S, Temizhan A, et al. Usefulness of Fragmented QRS Complex to Predict Arrhythmic Events and Cardiovascular Mortality in Patients With Noncompaction Cardiomyopathy. Am J Cardiol. 2016 May 1;117(9):1516–23.

8. Correia E, Rodrigues B, Santos L, Faria R, Ferreira P, Gama P, et al. Noncompaction of the ventricular myocardium: characterization and follow-up of an affected population. Rev Port Cardiol. 2011 Mar;30(3):323–31.

9. Enríquez R A, Baeza V R, Gabrielli N L, Córdova A S, Castro G P. Non compaction cardiomyopathy: a series of 15 cases. Revista médica de Chile. 2011 Jul;139(7):864–71.

10. Greutmann M, Mah ML, Silversides CK, Klaassen S, Attenhofer Jost CH, Jenni R, et al. Predictors of adverse outcome in adolescents and adults with isolated left ventricular noncompaction. Am J Cardiol. 2012 Jan 15;109(2):276–81.

11. Habib G, Charron P, Eicher J-C, Giorgi R, Donal E, Laperche T, et al. Isolated left ventricular non-compaction in adults: clinical and echocardiographic features in 105 patients. Results from a French registry. Eur J Heart Fail. 2011 Feb;13(2):177–85.

12. Ivanov Alexander, Dabiesingh Devindra S., Bhumireddy Geetha P., Mohamed Ambreen, Asfour Ahmed, Briggs William M., et al. Prevalence and Prognostic Significance of Left Ventricular Noncompaction in Patients Referred for Cardiac Magnetic Resonance Imaging. Circulation: Cardiovascular Imaging. 2017 Sep 1;10(9):e006174.

13. Kawasaki T, Azuma A, Taniguchi T, Asada S, Kamitani T, Kawasaki S, et al. Heart rate variability in adult patients with isolated left ventricular noncompaction. Int J Cardiol. 2005 Mar 10;99(1):147–50.

14. Li Shijie, Zhang Ce, Liu Nana, Bai Hui, Hou Cuihong, Wang Jizheng, et al. Genotype‐Positive Status Is Associated With Poor Prognoses in Patients With Left Ventricular Noncompaction Cardiomyopathy. Journal of the American Heart Association. 2018 Oct 16;7(20):e009910.

15. Lofiego C, Biagini E, Pasquale F, Ferlito M, Rocchi G, Perugini E, et al. Wide spectrum of presentation and variable outcomes of isolated left ventricular non-compaction. Heart. 2007 Jan;93(1):65–71.

16. Murphy RT, Thaman R, Blanes JG, Ward D, Sevdalis E, Papra E, et al. Natural history and familial characteristics of isolated left ventricular non-compaction. Eur Heart J. 2005 Jan;26(2):187–92.

17. Peters F, Khandheria BK, Botha F, Libhaber E, Matioda H, Dos Santos C, et al. Clinical outcomes in patients with isolated left ventricular noncompaction and heart failure. J Card Fail. 2014 Oct;20(10):709–15.

18. Ritter M, Oechslin E, Sütsch G, Attenhofer C, Schneider J, Jenni R. Isolated noncompaction of the myocardium in adults. Mayo Clin Proc. 1997 Jan;72(1):26–31.

19. Salazar-Mendiguchía J, González-Costello J, Oliveras T, Gual F, Lupón J, Manito N. Long-term Follow-up of Symptomatic Adult Patients With Noncompaction Cardiomyopathy. Revista Española de Cardiología (English Edition). 2019 Feb 1;72(2):169–71.

20. Sedaghat-Hamedani F, Haas J, Zhu F, Geier C, Kayvanpour E, Liss M, et al. Clinical genetics and outcome of left ventricular non-compaction cardiomyopathy. Eur Heart J. 2017 Dec 7;38(46):3449–60.

21. Steffel J, Hürlimann D, Namdar M, Despotovic D, Kobza R, Wolber T, et al. Long-term follow-up of patients with isolated left ventricular noncompaction: role of electrocardiography in predicting poor outcome. Circ J. 2011;75(7):1728–34.

22. Stöllberger C, Wegner C, Finsterer J. Left ventricular hypertrabeculation/noncompaction, cardiac phenotype, and neuromuscular disorders. Herz. 2019 Nov;44(7):659–65.

23. Tian T, Liu Y, Gao L, Wang J, Sun K, Zou Y, et al. Isolated left ventricular noncompaction: clinical profile and prognosis in 106 adult patients. Heart Vessels. 2014 Sep;29(5):645–52.

24. Tian T, Yang K-Q, Mao Y, Zhou L-L, Wang L-P, Xiao Y, et al. Left Ventricular Noncompaction in Older Patients. Am J Med Sci. 2017;354(2):140–4.

25. Gaye ND, Ngaïdé AA, Bah MB, Babaka K, Mbaye A, Abdoul K. Non-compaction of left ventricular myocardium in sub-Saharan African adults. Heart Asia. 2017;9(2):e010884.

26. Mazurkiewicz Ł, Petryka J, Śpiewak M, Miłosz-Wieczorek B, Małek ŁA, Jasińska A, et al. Clinical and prognostic relevancy of left ventricular trabeculation assessed by cardiac magnetic resonance in patients with dilated cardiomyopathy. Kardiologia Polska (Polish Heart Journal). 2017;75(8):794–803.

27. Stämpfli SF, Erhart L, Hagenbuch N, Stähli BE, Gruner C, Greutmann M, et al. Prognostic power of NT-proBNP in left ventricular non-compaction cardiomyopathy. Int J Cardiol. 2017 Jun 1;236:321–7.

28. Stanton C, Bruce C, Connolly H, Brady P, Syed I, Hodge D, et al. Isolated left ventricular noncompaction syndrome. Am J Cardiol. 2009 Oct 15;104(8):1135–8.

29. van Waning JI, Caliskan K, Hoedemaekers YM, van Spaendonck-Zwarts KY, Baas AF, Boekholdt SM, et al. Genetics, Clinical Features, and Long-Term Outcome of Noncompaction Cardiomyopathy. J Am Coll Cardiol. 2018 Feb 20;71(7):711–22.

30. Amzulescu M-S, Rousseau MF, Ahn SA, Boileau L, de Meester de Ravenstein C, Vancraeynest D, et al. Prognostic Impact of Hypertrabeculation and Noncompaction Phenotype in Dilated Cardiomyopathy: A CMR Study. JACC: Cardiovascular Imaging. 2015 Aug 1;8(8):934–46.
